# The Therapeutic Effect of Nordic Walking on Freezing of Gait in Parkinson's Disease: A Pilot Study

**DOI:** 10.1155/2019/3846279

**Published:** 2019-12-27

**Authors:** Agata Wróblewska, Agata Gajos, Urszula Smyczyńska, Andrzej Bogucki

**Affiliations:** ^1^Department of Extrapyramidal Diseases, Medical University of Łódź, Pomorska 251, 92-213 Łódź, Poland; ^2^Department of Biostatistics and Translational Medicine, Medical University of Łódź, Mazowiecka 15, 92-215 Łódź, Poland

## Abstract

*Introduction*. The effectiveness of the currently utilized therapies for FoG is limited. Several studies demonstrated a beneficial impact of Nordic walking (NW) on several gait parameters in Parkinson's disease, but only one paper reported reduction of freezing. *Research Question*. In the present study, the question is whether NW is an effective therapeutic intervention in FoG.

**Methods:**

Twenty PD subjects trained NW for 12 weeks, with a frequency of twice per week. Each session lasted about 60 minutes. Twenty patients in the control group did not use any form of physiotherapy (no-intervention group). Freezing of Gait Questionnaire (FOGQ), the Timed Up and Go (TUG) test, and the Provocative Test for Freezing and Motor Blocks (PTFMB) were performed at baseline, immediately after the end of NW program, and three months later.

**Results:**

The results of FOGQ, TUG, and total PTFMB revealed significant improvement after completing the exercise program, and this effect persisted at follow-up. The results of the PTFMB subtests showed a different effect of NW on particular subtypes of FoG. Start hesitation, sudden transient blocks that interrupt gait, and blocks on turning improved considerably, while motor blocks, when walking through narrow space and on reaching the target, did not respond to NW training. *Significance*. The results show, for the first time, that FoG during turning and step initiation, two most common forms of this gait disorder, has been significantly reduced by NW training. Different responses of particular subtypes of FoG to NW probably reflect their different pathophysiologies.

**Conclusions:**

The present study showed that NW training had a beneficial effect on FOG in PD and that the achieved improvement is long-lasting. Future research should clarify whether the observed improvement limited to FoG triggered by only some circumstances reflects different pathomechanisms of FoG subtypes.

## 1. Introduction

Freezing of gait (FoG) is a disabling phenomenon usually observed in the more advanced stages of Parkinson's disease (PD) [1–3]. According to the proposed definition, FoG is a “brief, episodic absence or marked reduction of forward progression of feet despite the intention to walk” [4]. FoG leads to impaired mobility, significantly increases the risk of falling over, and interferes with daily activities [5, 6]. Moreover, it has a significantly negative impact on the quality of life of PD patients [7, 8].

Pathophysiology of FoG is still poorly understood [9, 10]. Limited effectiveness of the currently utilized therapies for FoG most probably reflects the complexity of pathomechanism of this phenomenon. Optimization of pharmacotherapy (especially the adjustment of dopaminergic treatment), external cueing, deep brain stimulation, and different forms of physiotherapy are among the proposed treatment options [11].

Nordic walking (NW) is being increasingly used in physical therapy of different conditions, including PD, in recent years. The majority of studies demonstrated a beneficial impact of NW on gait in PD as shown by recently published two systematic reviews [12, 13]. The related NW training improvement was observed in several gait parameters including step length, walking velocity, gait pattern [14–17], temporal organization of gait [18], and postural abilities [17, 19].

Data on the impact of NW on FoG are very scarce. Only one study reported less freezing in the NW group [16]. However, to measure the severity of FoG, the item 14 of UPDRS, part II was used, which is not a precise tool, and it takes into account only the patient's own observations. The aim of the present study was to assess the impact of NW training on FoG using tools dedicated for the evaluation of freezing in PD.

## 2. Methods

### 2.1. Participants

Patients were screened for FoG and study eligibility via medical records review. Forty outpatients diagnosed with PD according to UK PD Brain Bank [20] criteria who suffered from FoG episodes during ON state were recruited into the study. All of them experienced FoG incidents despite optimized treatment with oral drugs. Polypharmacotherapy was used in all patients. All subjects were treated with different formulations of levodopa and ropinirole, and in addition, some of them also received other antiparkinsonian drugs. The mean levodopa equivalent daily dose was 980.3 ± 262.9 for NW and 1057.1 ± 344.6 for the control group (n.s.).

None of the patients had significant motor fluctuations. Inclusion criteria comprised Hoehn and Yahr [21] stages II to III, stable pharmacotherapy for at least 4 weeks before the study began, sufficient general health condition for the training intervention, and no previous experience with NW. Patients did not participate in any form of physiotherapy or any regular sport programs for at least four months before enrollment. The study was approved by the Ethics Committee at the Medical University of Łódź, and written informed consent was obtained from each subject.

### 2.2. Nordic Walking Training

On the first visit, participants were randomized into the NW or no-intervention group. Randomisation was conducted by using the online service (http://www.randomizer.org). The main NW training period was preceded by three preliminary sessions to familiarize subjects with NW technique. Then, the patients were trained outdoor for 12 weeks, with a frequency of twice per week. They were accompanied by physiotherapist, a qualified NW instructor during all sessions. Each session lasted about 60 minutes and included three phases: (a) initial stretching and warming up, (b) practicing NW competence, and (c) final stretching and cooling down.

During the entire NW training period, subjects did not participate in any other training program or physiotherapy. The doses of antiparkinsonian medications were kept unchanged. Patients in the control group did not use any form of physiotherapy (no-intervention group). Patients in both groups were advised not to change their current lifestyle, and all previously practiced forms of leisure activity were allowed during the study.

### 2.3. Clinical Assessment

Unified Parkinson's Disease Rating Scale, Part III (UPDRS III) and Hoehn and Yahr scale (H-Y) were assessed at baseline. FoG was evaluated: (1) at baseline, (2) immediately after the end of NW exercise program, and (3) three months later—follow-up (NW group only). All evaluations were performed while patients were in the medication ON state. The following tests were performed:

  Freezing of Gait Questionnaire (FOGQ) consists of 6 items, related to FoG and walking, with a choice of 5 answers, scored from 0 to 4. The maximum score adds up to 24 points with a higher score indicating more severe FoG [22].
  Timed Up and Go test (TUG) measures the time needed by the patient to perform sequential locomotor tasks that incorporate walking and turning [23].
  The Provocative Test for Freezing and Motor Blocks (PTFMB). In PTFMB, the following are scored: start hesitation, sudden transient blocks that interrupt gait, motor blocks on turning, motor blocks on reaching a target, and motor blocks when walking through narrow space. The tasks are rated as not observed (0 = no) or observed (1 = yes). The maximum score for freezing is 5 if all modalities of FoG were observed [24, 25].

### 2.4. Statistical Analysis

Statistical analysis of data was performed with the use of Statistics and Python statistical packages. In all tests, a *p*-value below 0.05 was considered significant. First, the NW and control groups were compared in order to detect potential differences in baseline characteristics: age, duration, and severity of disease. The proportion of males and females in the intervention and control groups were compared by Fisher's exact test. Age was compared by *t*-test after its assumption was verified (normality of distribution by the Shapiro–Wilk test and equality of variances by Bartlett's test). The differences in duration of the disease in two groups of patients were assessed by the Mann–Whitney test because the assumption of *t*-test was violated in this case.

Proportions of patients with II and III stages of Parkinson's disease in 2 groups were compared by Mood's median test. The analysis of results of the intervention in the form of Nordic walking training was performed in two stages:

1. Assessment of NW effectiveness directly after the last training session with respect to the no-intervention group;
2. Assessment of persistence of the effect in the NW group.

In the first step, 2 groups–2 timepoints analysis of FOGQ, TUG results, and the total score from PTFMB was performed with the use of generalized linear models (GLMs) with repeated measurements. Additionally, disease duration was included as an interfering factor in models, since the difference in these baseline characteristics of the groups proved to be statistically significant (Table 1). The model also allowed the analysis of 2-factor interactions: between the timepoint (before/after intervention) and the group (intervention/control) and between the timepoint and disease duration.

Constancy of the NW training effect was assessed by comparison of FOGQ and TUG results and the total score in PTFMB between 3 timepoints in the intervention group: before any NW session started, after the whole of the NW training period, and at follow-up 3 months later. The Friedman test was used for this comparison because again the assumptions for the parametric test (ANOVA) were not fulfilled. When the result was significant, the Friedman test was followed by post hoc comparison by the Nemenyi test.

In case of PTFMB, whose components represent the presence or absence of particular FOG symptoms, statistical tests were applied to find differences in frequency of those symptoms between the groups or timepoints. When 2 distinct, independent groups of patients (NW group and control group) were considered, the frequencies of symptoms occurrence were compared by *χ*^2^ test for contingency tables. Whenever the samples were dependent (2 different timepoints in the same group), the McNemara test was used.

## 3. Results

The NW group consisted of 8 females and 12 males, with a mean age of 72.1 ± 7.5 and a mean disease duration of 5.2 ± 1.1 years. The mean age of 11 females and 9 males in the control group was 67.6 ± 6.6 years, and the mean disease duration was 6.0 ± 1.2 years. The difference in age between the groups was on the verge of statistical significance (*p*=0.051). The duration of disease was significantly longer in the control group (*p*=0.04); therefore, it was included in further analysis as a cofactor; however, this difference on average equaled only 0.8 year.

In the NW group, 9 patients were in H-Y stage II and 11 in stage III. In the control group, 11 and 9 subjects presented H-Y stage II and III, respectively. The difference in proportion of stage II and III in both groups was statistically insignificant (*p*=0.75). The results of UPDRS III motor section obtained at baseline in the NW and control groups did not differ significantly (32.7 ± 6.9 vs. 32.0 ± 7.7, respectively, *p*=0.76).

All baseline characteristics of recruited patients are summarized in Table 1. All patients completed the entire training period, and the adherence to the protocol was 93%.

### 3.1. Freezing of Gait Questionnaire

**Figure 1:**
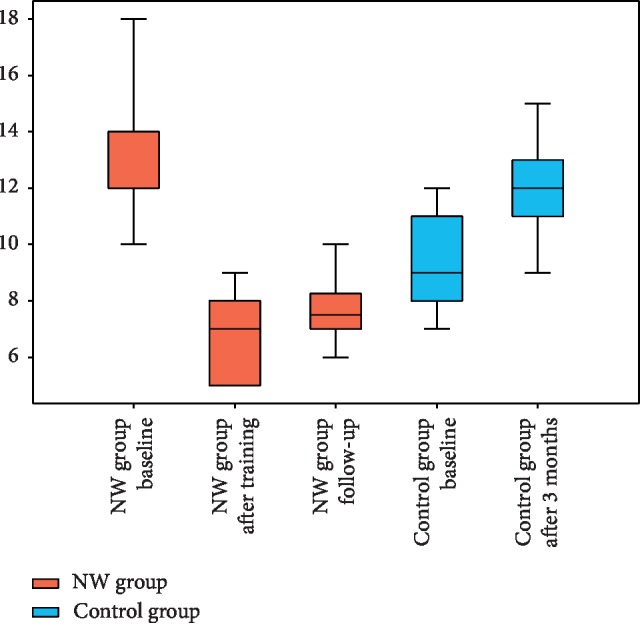
Freezing of Gait Questionnaire scores in the NW and control groups at different timepoints. In all boxplots, the median is presented together with interquartile range; whiskers represent the 5th and 95th centile.

The changes in FOGQ results after the NW training period in both groups were analysed by GLM that achieved statistical significance of *p* < 0.0001. After completing the three-month NW training, considerable improvement of FoG was achieved in the intervention group while the second evaluation of control subjects revealed significant deterioration (Figure 1, Table 2). Those changes are due to both group assignment and the timepoint as shown by *p* < 0.0001 for interaction between the group and timepoint (Table 3). What was significant was the interaction rather than the timepoint alone (training status) because the baseline FOGQ score was much higher in the intervention group. The above result has been corrected for the difference in disease duration that in case was also a significant factor (*p*=0.01) affecting the FOGQ score.

The analysis of persistence of effects of training in the NW group (Table 4) revealed significant differences in FOGQ scores between the timepoints with *p* < 0.0001 in the Friedman test. The post hoc Nemenyi test showed that differences between the baseline score and those obtained directly after training and at follow-up are significant (*p*=0.001 in both cases) while the change in scores during the 3 month follow-up period is insignificant (*p* < 0.14).

### 3.2. Timed Up and Go Test

**Figure 2:**
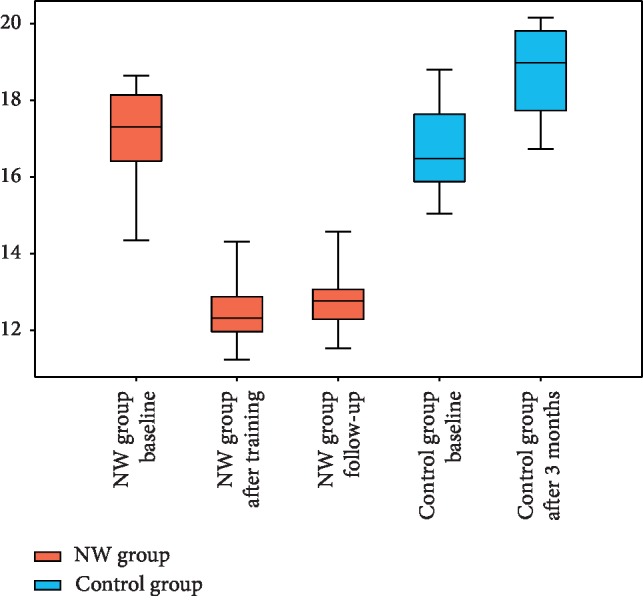
Timed Up and Go Test results in the NW and control groups at different timepoints.

The results obtained by patients in TUG (Figure 2, Table 5) were also assessed by the means of generalized linear model (*p* < 0.0001). In this case, two significant factors were identified (Table 2): group assignment (*p* < 0.0001) and interaction between the group and timepoint (*p* < 0.0001). Again, significant improvement was achieved in the NW group, while the results in the no-intervention control group deteriorated.

The analysis of persistency of the NW training effect again showed significance of changes of TUG in the intervention group (*p* < 0.0001 in the Friedman test). All 2-timepoint post hoc comparisons indicated statistically significant differences; however, during the follow-up period, TUG increased only by 0.4 while the decrease associated with training was 10 times greater equaling 4.3. Statistical significance in comparison between end of training and follow-up should be associated with the fact that slight increase of TUG was observed in every single patient rather than with magnitude of the difference.

### 3.3. The Provocative Test for Freezing and Motor Blocks

At baseline, the total score of PTFMB showed more severe FOG in the NW group than in the control one, equaling 3.5 and 2.8, respectively. The total score was significantly reduced to only 0.6 at the end of the NW program, while in the control group, it increased to 3.1. In the analysis of the first two timepoints in both groups, the PTFMB score was shown dependent on group assignment (*p* < 0.0001) and the group-timepoint interaction term (*p* < 0.0001), but not on the duration of the disease (*p*=0.22), according to GLM analysis (Table 3). At follow-up, the PTFMB total score in the intervention group remained low at the level of 0.7 with no significant difference with respect to measurement at the end of the training period (Table 4).

**Figure 3:**
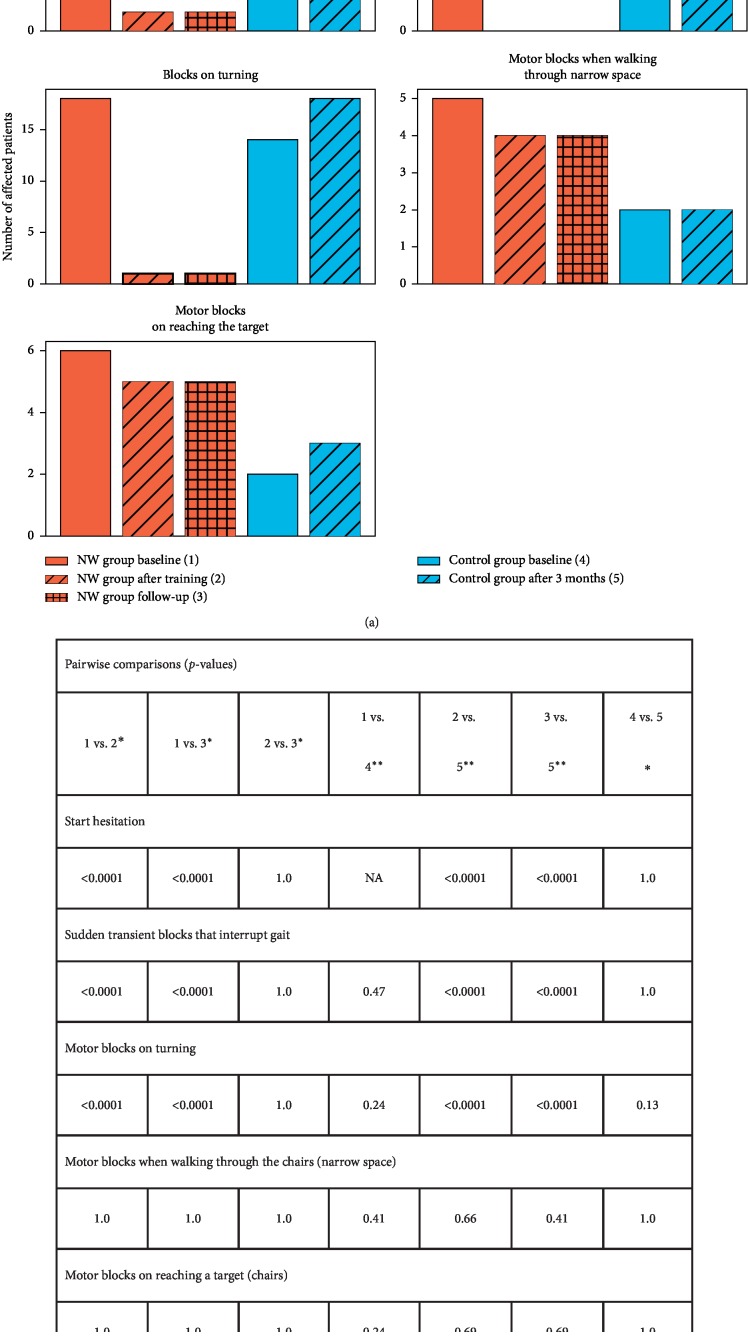
Results of the Provocative Test for Motor Blocks. (a) Number of patients in each group affected by a particular symptom at different timepoints; significant differences are marked in red. (b) Results of pairwise comparisons between the groups/timepoints; timepoint numbering is explained in legend.

The results of the five tasks included in the PTFMB test were also evaluated separately (Figure 3(a)). The numbers of subjects demonstrating individual forms of freezing were assessed. Considerable improvement was observed in start hesitation, sudden transient blocks that interrupt gait, and motor blocks on turning in the NW group. All subjects in the NW group initially showed start hesitation and sudden gait interruptions. The second examination revealed start hesitation in only 2 patients, and gait blocks were no longer observed in any of the 20 patients. Significant reduction (from 18 to 1) in the number of subjects with turning hesitation was also found. The beneficial effect of NW training was maintained for the next three months. All of the above results were shown to be statistically significant with *p* < 0.0001 by tests listed in Figure 3(b). Freezing when walking through narrow space and on reaching a target did not respond to NW. No significant differences between both evaluations were found in the control group.

## 4. Discussion

To our knowledge, this is the first report of improved FoG in individuals with PD as a result of a NW training intervention.

Although FoG is predominantly present in more advanced PD with the incidence of 80% in case of 20 years' disease duration [26], it was also observed in L-Dopa naïve subjects at the early stage of the disease [27]. The presence of FoG significantly increases the risk of falls [28].

FoG may present as (1) shuffling forward with small steps, (2) trembling in place with steps blocked and alternating rapid knee movements, and (3) as complete akinesia. Incidents of FoG occur most commonly during turning and gait initiation (turning or start hesitation). Other triggering circumstances are passing through narrow spaces and reaching a destination (tight quarters and destination hesitation, respectively). Walking along a straight line in open space may also be interrupted by FoG [29]. It remains unexplained whether this different symptomatology reflects only the severity of the disease or whether distinct pathomechanisms are responsible for particular phenomenological subtypes of FoG.

Pathophysiology of FoG is poorly understood. The proposed hypothetical mechanisms of FoG are, among others, the disturbed central drive and automaticity, abnormality of gait pattern generation, and rhythm formation as well as frontal executive dysfunction [30]. In patients experiencing a wearing-off phenomena, the incidence of FoG usually occurs in the off state and they respond to dopaminergic treatment including levodopa and dopamine agonists [31]. With advancing disease pharmacological control FoG becomes less effective, although true L-dopa-resistant FoG is relatively rare.

There are limited nonpharmacological treatment options including some trick movements or strategies that can be helpful for alleviating FoG. Rhythmic auditory and visual cues generated by various devices and stick projecting a laser line on the floor in front of a patient were found to be effective in FoG [32]; however, the duration of the improvement was limited to the time when cueing was used.

In the recently proposed therapeutic algorithm, physiotherapy is recommended for treatment of both mild and troublesome FoG [33]. The effectiveness of several complementary interventions, including tango, Irish dance, Tai Chi, theatre training, and NW, in the treatment of motor function in PD was studied.

Tango dancing improves walking in PD patients [34–36]. Some studies using FOGQ showed that tango training programs were also effective in improving FoG [37, 38], but it was not confirmed by a 2-year prospective pilot study [39]. Finally, the results of a systematic review and meta-analysis published in 2015 did not support the hypothesis that tango is an effective intervention for FoG in PD [40].

A randomized controlled study comparing Irish set dancing with standard physiotherapy revealed that only the first of these interventions significantly reduced FoG as demonstrated by results of modified FOGQ [41].

Tai Chi has the beneficial effect on balance and mobility in PD [42], and it is also effective in reducing falls incidence [43–45]; however, the effect of Tai Chi on FoG was not studied.

In turn, the 3-year active theater training program allows patients to get well-being without noticeable influence on motor functions (gait was not evaluated) [46, 47].

The impact on FoG of any other comparative intervention in PD had not been studied as thoroughly as we did with NW. NW is a fitness marching with poles adapted from cross-country skiing. NW training activates the upper body and is a factor forcing better coordination between lower and upper limbs when walking. The beneficial impact of NW on several gait parameters in PD is well documented [12, 13]. However, only one study reported reduced FoG in the NW group [16], and it was revealed by the results of the item 14 of UPDRS, part II. It was suggested that NW can improve gait in PD because it is a form of external cueing that increases the rhythmicity of movements [18].

Basal ganglia (BG) with conjunction of supplementary motor area (SMA) run automatic movements. Depending on what motor skill is to be performed, the appropriate movement amplitude is preset (“motor set”), and it is then maintained thanks to the functional connection of BG with SMA. BG are responsible for generating timing cues and in this way control the execution of subsequent movements that make up a more complex movement. It was suggested that the impaired BG function in PD leads to abnormalities of motor set and motor cues and in consequence the initiation deficits and reduced step length. Such mechanism may be responsible for incidents of gait blocking [48]. So, NW can be considered as the kind of cueing in which upper limb movements are the source of external information enabling the normalization of speed and amplitude of leg movements while walking.

It should be noted that in contrast to gait asymmetry, which is more severe in patients with FoG and additionally worsens in the off state, asymmetry of upper extremity rhythmic movements is similar in PD subjects with and without FoG, and it is not influenced by on and off fluctuations [49]. It allows the use of rhythmic movements of the upper limbs during NW as a source of external sensory information.

Performing the secondary motor or cognitive task while walking (dual tasking) could provoke FoG [50], and this phenomenon can be explained by impaired movement automaticity. It may be suggested that during NW training, rhythmic upper limb movements focus patients' attention on walking and enable them to rely on the automaticity of gait to a lesser extent.

The quantitative assessment of FoG is difficult due to its paroxysmal, irregular occurrence, different duration of freezing episodes, and their sensitivity to environmental triggers such as emotional factors [51]. Questionnaires adequately assess the frequency and severity of FoG episodes in a home environment while tests allow the clinician to observe the occurrence of freezing in standard conditions. FOGQ used in our study originates from recommended questionnaires, and TUG was also selected from recommended tests with confirmed reliability for the assessment of gait abnormalities in PD [52]. In addition, we used PTFMB that makes it possible to assess the occurrence of FoG triggered by different circumstances.

The significant beneficial effect of NW on FoG was revealed by the results of FOGQ and TUG as well as by total results of PTFMB. And what is at least equally important is that this therapeutic effect was maintained for the next three months. Our results confirm the previously published data [15] showing that the gait improvement produced by NW may persist for several months.

The results of the PTFMB subtests were just as surprising as intriguing, as they showed a different effect of NW on particular subtypes of FoG. Start hesitation and sudden transient blocks were present in all patients in the NW group, and blocks on turning were observed in 18 of 20 subjects. These three forms of FoG showed considerable and long-lasting improvement. This finding is of great importance from a practical point of view because PD patients experience FoG incidents most often during turning and step initiation.

NW did not influence motor blocks when walking through narrow space and on reaching the target; they were present in 6 and 5 subjects, respectively. As it was said before, we know too little about pathophysiology of FoG to speculate whether our results should be interpreted as confirming the existence of separate mechanisms responsible for FoG subtypes. It should be noted, however, that the recently published study revealed the same efficacy of STN-DBS in all types of FOG provoked by different circumstances [53].

We are aware of methodological limitations of our study, and therefore, we believe that it should be considered a pilot study.

Firstly, our sample size was relatively small, especially given the variability in individuals with PD, but it was similar to those of most studies on complementary interventions in PD. Into the study, we included patients Hoehn and Yahr stages 2-3 only to obtain a homogenous group of patients with a small variation in the severity of motor symptoms.

Secondly, the no-intervention arm was used as the control group, as we encountered difficulties in recruiting patients for the usual walking control group and in obtaining their availability for the duration of the study. Therefore, the placebo effect cannot be excluded. In addition, we were not able to perform the follow-up evaluation in the control group due to drop-out of patients.

Thirdly, we did not address the intensity, frequency, and duration of the NW training in our protocol. We used NW procedures similar to those previously proposed by other authors, but it cannot be ruled out that these parameters were not optimal.

Despite the limitations, our results are promising enough to expand this pilot study to a larger study. FoG is a complex clinical phenomenon, and protocols and tools used in future studies should enable assessment of the effect of NW on FoG subtypes in particular individuals and triggered by different circumstances.

## 5. Conclusion

The present study shows that NW training has a beneficial effect on FOG in PD and that the achieved improvement is long-lasting. Future research should clarify whether the observed improvement limited to FoG triggered by only some circumstances reflects different pathomechanisms of FoG subtypes.

## Figures and Tables

**Table 1:** Baseline patients' characteristics.

	All patients	NW group	Control	*p* (in tests for differences between 2 groups)
Number of patients	40	20 (8 males)	20 (9 males)	0.53 (Fisher's exact test)
Age (years), mean ± SD (range)	69.8 ± 7.3 (58–84)	72.1 ± 7.5 (58–84)	67.6 ± 6.6 (58–82)	0.051 (*t*-test for independent groups)
Duration of disease (years), mean ± SD (range)	5.6 ± 1.2 (4–8)	5.2 ± 1.1 (4–7)	6.0 ± 1.2 (4–8)	0.04 (Mann–Whitney test)
H-Y	II in 20 patients III in 20 patients	II in 9 patients, III in 11 patients	II in 11 patients III in 9 patients	0.75 (Mood's median test)
UPDRS III (range)	32.3 ± 7.3 (17–47)	32.7 ± 6.9 (21–46)	32.0 ± 7.7 (17–47)	0.76 (*t*-test for independent groups)

**Table 2:** Freezing of Gait Questionnaire.

	NW group			Control group	
	Baseline (1)	After NW program (2)	Follow-up (3)	Baseline (4)	After 3 months (5)
Mean ± SD (range)	13.8 ± 2.3 (10–19)	7.1 ± 1.7 (5–11)	7.9 ± 1.5 (5–11)	9.3 ± 1.8 (7–12)	12.0 ± 1.9 (8–15)
Median (interquartile range)	14 (12–14)	7 (5–8)	7.5 (7-8.3)	9 (8–11)	12 (11–13)

**Table 3:** Factors affecting FOGQ, TUG results, and the PTFMB total score before and after NW training.

	FOGQ		TUG		PTFMB total	
	SS	*p*	SS	*p*	SS	*p*
Intercept	124.67	<0.0001	616.44	<0.0001	10.35	0.0003
Group	2.20	0.52	112.58	<0.0001	12.76	<0.0001
Disease duration	45.49	0.01	7.15	0.15	1.02	0.22
Timepoint	4.81	0.06	1.60	0.19	0.41	0.10
Timepoint ∗ group	391.59	<0.0001	190.47	<0.0001	46.21	<0.0001
Timepoint ∗ disease duration	0.24	0.66	0.03	0.86	0.20	0.24

SS: sum of squares for the effect (variance explained by the effect).

**Table 4:** Persistence of NW training effects assessed by FOGQ and TUG (*p* values from the Nemenyi post hoc test).

	Before NW vs after NW	Before NW vs at follow-up	After NW vs follow-up
FOGQ	0.001	0.001	0.14
TUG	0.001	0.012	0.003
PTFMB total score	0.001	0.001	0.90

**Table 5:** Timed Up and Go Test.

	NW group			Control group	
	Baseline (1)	After NW program (2)	Follow-up (3)	Baseline (4)	After 3 months (5)
Mean ± SD (range)	17.2 ± 1.4 (13.9–18.9)	12.6 ± 1.4 (10.6–17.4)	12.9 ± 1.5 (10.6–18.2)	16.6 ± 1.5 (12.4–18.9)	18.6 ± 1.5 (14.8–20.7)
Median (interquartile range)	17.3 (16.4–18.1)	12.3 (12.0–12.9)	12.8 (12.3–13.1)	16.5 (15.9–17.6)	19.0 (17.8–19.8)

## Data Availability

The outcome data used to support the findings of this study are available from the corresponding author upon request.

## References

[1] Giladi N., Treves T. A., Simon E. S. (2001). Freezing of gait in patients with advanced Parkinson’s disease. *Journal of Neural Transmission*.

[2] Perez-Lloret S., Negre-Pages L., Damier P. (2014). Prevalence, determinants, and effect on quality of life of freezing of gait in Parkinson disease. *JAMA Neurology*.

[3] Contreras A., Grandas F. (2012). Risk factors for freezing of gait in Parkinson’s disease. *Journal of the Neurological Sciences*.

[4] Nutt J. G., Bloem B. R., Giladi N., Hallett M., Horak F. B., Nieuwboer A. (2011). Freezing of gait: moving forward on a mysterious clinical phenomenon. *The Lancet Neurology*.

[5] Bloem B. R., Hausdorff J. M., Visser J. E., Giladi N. (2004). Falls and freezing of gait in Parkinson’s disease: a review of two interconnected, episodic phenomena. *Movement Disorders*.

[6] Kerr G. K., Worringham C. J., Cole M. H., Lacherez P. F., Wood J. M., Silburn P. A. (2010). Predictors of future falls in Parkinson disease. *Neurology*.

[7] Moore O., Peretz C., Giladi N. (2007). Freezing of gait affects quality of life of peoples with Parkinson’s disease beyond its relationships with mobility and gait. *Movement Disorders*.

[8] Walton C. C., Shine J. M., Hall J. M. (2015). The major impact of freezing of gait on quality of life in Parkinson’s disease. *Journal of Neurology*.

[9] Danoudis M., Iansek R., Simpson P. (2012). Freezing of gait in Parkinson’s disease: further insights into pathophysiological mechanisms. *Parkinsonism & Related Disorders*.

[10] Snijders A. H., Takakusaki K., Debu B. (2016). Physiology of freezing of gait. *Annals of Neurology*.

[11] Alves Da Rocha P., McClelland J., Morris M. E. (2015). Complementary physical therapies for movement disorders in Parkinson’s disease: a systematic review. *European Journal of Physical and Rehabilitation Medicine*.

[12] Bombieri F., Schena F., Pellegrini B., Barone P., Tinazzi M., Erro R. (2017). Walking on four limbs: a systematic review of Nordic Walking in Parkinson disease. *Parkinsonism & Related Disorders*.

[13] Cugusi L., Manca A., Dragone D. (2017). Nordic walking for the management of people with Parkinson disease: a systematic review. *PM&R*.

[14] Monteiro E. P., Franzoni L. T., Cubillos D. M. (2017). Effects of Nordic walking training on functional parameters in Parkinson’s disease: a randomized controlled clinical trial. *Scandinavian Journal of Medicine & Science in Sports*.

[15] van Eijkeren F. J. M., Reijmers R. S. J., Kleinveld M. J., Minten A., Bruggen J. P. T., Bloem B. R. (2008). Nordic walking improves mobility in Parkinson’s disease. *Movement Disorders*.

[16] Reuter I., Mehnert S., Leone P., Kaps M., Oechsner M., Engelhardt M. (2011). Effects of a flexibility and relaxation programme, walking, and nordic walking on Parkinson’s disease. *Journal of Aging Research*.

[17] Cugusi L., Solla P., Serpe R. (2015). Effects of a Nordic Walking program on motor and non-motor symptoms, functional performance and body composition in patients with Parkinson’s disease. *NeuroRehabilitation*.

[18] Warlop T., Detrembleur C., Lopez M. B., Stoquart G., Lejeune T., Jeanjean A. (2017). Does Nordic Walking restore the temporal organization of gait variability in Parkinson’s disease?. *Journal of NeuroEngineering and Rehabilitation*.

[19] Gougeon M.-A., Zhou L., Nantel J. (2017). Nordic Walking improves trunk stability and gait spatial-temporal characteristics in people with Parkinson disease. *NeuroRehabilitation*.

[20] Hughes A. J., Daniel S. E., Kilford L., Lees A. J. (1992). Accuracy of clinical diagnosis of idiopathic Parkinson’s disease: a clinico-pathological study of 100 cases. *Journal of Neurology, Neurosurgery & Psychiatry*.

[21] Hoehn M. M., Yahr M. D. (1967). Parkinsonism: onset, progression, and mortality. *Neurology*.

[22] Giladi N., Shabtai H., Simon E. S., Biran S., Tal J., Korczyn A. D. (2000). Construction of freezing of gait questionnaire for patients with Parkinsonism. *Parkinsonism & Related Disorders*.

[23] Morris S., Morris M. E., Iansek R. (2001). Reliability of measurements obtained with the timed “up & Go” test in people with Parkinson disease. *Physical Therapy*.

[24] Thomas M., Jankovic J., Suteerawattananon M. (2004). Clinical gait and balance scale (GABS): validation and utilization. *Journal of the Neurological Sciences*.

[25] Protas E. J., Mitchell K., Williams A., Qureshy H., Caroline K., Lai E. C. (2005). Gait and step training to reduce falls in Parkinson’s disease. *NeuroRehabilitation*.

[26] Hely M. A., Reid W. G. J., Adena M. A., Halliday G. M., Morris J. G. L. (2008). The Sydney multicenter study of Parkinson’s disease: the inevitability of dementia at 20 years. *Movement Disorders*.

[27] Giladi N., McDermott M. P., Fahn S. (2001). Freezing of gait in PD: prospective assessment in the DATATOP cohort. *Neurology*.

[28] Okuma Y., Silva de Lima A. L., Fukae J., Bloem B. R., Snijders A. H. (2018). A prospective study of falls in relation to freezing of gait and response fluctuations in Parkinson’s disease. *Parkinsonism & Related Disorders*.

[29] Okuma Y. (2014). Practical approach to freezing of gait in Parkinson’s disease. *Practical Neurology*.

[30] Okuma Y. (2014). Freezing of gait and falls in Parkinson’s disease. *Journal of Parkinson’s Disease*.

[31] Schaafsma J. D., Balash Y., Gurevich T., Bartels A. L., Hausdorff J. M., Giladi N. (2003). Characterization of freezing of gait subtypes and the response of each to levodopa in Parkinson’s disease. *European Journal of Neurology*.

[32] Ginis P., Nackaerts E., Nieuwboer A., Heremans E. (2018). Cueing for people with Parkinson’s disease with freezing of gait: a narrative review of the state-of-the-art and novel perspectives. *Annals of Physical and Rehabilitation Medicine*.

[33] Nonnekes J., Snijders A. H., Nutt J. G., Deuschl G., Giladi N., Bloem B. R. (2015). Freezing of gait: a practical approach to management. *The Lancet Neurology*.

[34] Hackney M., Earhart G. (2009). Effects of dance on movement control in Parkinson’s disease: a comparison of Argentine tango and American ballroom. *Journal of Rehabilitation Medicine*.

[35] Hackney M. E., Earhart G. M. (2010). Effects of dance on gait and balance in Parkinson’s disease: a comparison of partnered and nonpartnered dance movement. *Neurorehabilitation and Neural Repair*.

[36] McNeely M. E., Mai M. M., Duncan R. P., Earhart G. M. (2015). Differential effects of tango versus dance for PD in Parkinson disease. *Front Aging Neurosci*.

[37] Duncan R. P., Earhart G. M. (2012). Randomized controlled trial of community-based dancing to modify disease progression in Parkinson disease. *Neurorehabilitation and Neural Repair*.

[38] Hackney M. E., Kantorovich S., Levin R., Earhart G. M. (2007). Effects of tango on functional mobility in Parkinsonʼs disease: a preliminary study. *Journal of Neurologic Physical Therapy*.

[39] Duncan R. P., Earhart G. M. (2014). Are the effects of community-based dance on Parkinson disease severity, balance, and functional mobility reduced with time? A 2-year prospective pilot study. *The Journal of Alternative and Complementary Medicine*.

[40] Lötzke D., Ostermann T., Büssing A. (2015). Argentine tango in Parkinson disease—a systematic review and meta-analysis. *BMC Neurology*.

[41] Volpe D., Signorini M., Marchetto A., Lynch T., Morris M. E. (2013). A comparison of Irish set dancing and exercises for people with Parkinson’s disease: a phase II feasibility study. *BMC Geriatrics*.

[42] Song R., Grabowska W., Park M. (2017). The impact of Tai Chi and Qigong mind-body exercises on motor and non-motor function and quality of life in Parkinson’s disease: a systematic review and meta-analysis. *Parkinsonism & Related Disorders*.

[43] Winser S. J., Tsang W. W., Krishnamurthy K., Kannan P. (2018). Does Tai Chi improve balance and reduce falls incidence in neurological disorders? A systematic review and meta-analysis. *Clinical Rehabilitation*.

[44] Gao Q., Leung A., Yang Y. (2014). Effects of Tai Chi on balance and fall prevention in Parkinson’s disease: a randomized controlled trial. *Clinical Rehabilitation*.

[45] Li F., Harmer P., Fitzgerald K. (2012). Tai chi and postural stability in patients with Parkinson’s disease. *New England Journal of Medicine*.

[46] Modugno N., Iaconelli S., Fiorlli M., Lena F., Kusch I., Mirabella G. (2010). Active theater as a complementary therapy for Parkinson’s disease rehabilitation: a pilot study. *The Scientific World Journal*.

[47] Mirabella G., De Vita P., Fragola M. (2017). Theatre is a valid add-on therapeutic intervention for emotional rehabilitation of Parkinson’s disease patients. *Parkinson’s Disease*.

[48] Iansek R., Danoudis M. (2016). Freezing of gait in Parkinson’s disease: its pathophysiology and pragmatic approaches to management. *Movement Disorders Clinical Practice*.

[49] Plotnik M., Giladi N., Balash Y., Peretz C., Hausdorff J. M. (2005). Is freezing of gait in Parkinson’s disease related to asymmetric motor function?. *Annals of Neurology*.

[50] Hallett M. (2008). The intrinsic and extrinsic aspects of freezing of gait. *Movement Disorders*.

[51] Barthel C., Mallia E., Debû B., Bloem B. R., Ferraye M. U. (2016). The practicalities of assessing freezing of gait. *Journal of Parkinson’s Disease*.

[52] Bloem B. R., Marinus J., Almeida Q. (2016). Measurement instruments to assess posture, gait, and balance in Parkinson’s disease: critique and recommendations. *Movement Disorders*.

[53] Barbe M. T., Barthel C., Chen L. (2018). Subthalamic nucleus deep brain stimulation reduces freezing of gait subtypes and patterns in Parkinson’s disease. *Brain Stimulation*.

